# Technostress in Spanish University Students: Validation of a Measurement Scale

**DOI:** 10.3389/fpsyg.2020.582317

**Published:** 2020-10-29

**Authors:** María Penado Abilleira, María Luisa Rodicio-García, María Paula Ríos-de-Deus, María José Mosquera-González

**Affiliations:** ^1^Facultad de Ciencias de la Salud, Universidad Isabel I, Burgos, Spain; ^2^Unidad de Investigación FORVI (Formación y Orientación para la Vida), Universidad de A Coruña, A Coruña, Spain

**Keywords:** technostress, university, students, questionnaire, Spanish validation

## Abstract

The increasingly widespread use of technology has led to the emergence of phenomena harmful to users such as technostress. Although technostress has already been studied in other contexts, it is still pending study in a university education environment, where the use of information and communication technologies is increasingly widespread. Thus, the objective of this study was to adapt a technostress questionnaire for Spanish university students based on an instrument that had been designed in a Chinese university teaching population. A total of 1,744 Spanish university students from face-to-face and online universities completed the adapted Spanish technostress scale. Factorial analyses suggested the elimination of two items from the original scale and a model made up of five factors that fit, as in the original scale, within the person-environment misfit theory. The reduced scale also showed good internal consistency for all the items and the five resulting factors. These results support the psychometric properties of the reduced technostress scale in university students, and their validity when offering a complete view of the phenomenon in Spain.

## Introduction

*Technostress* is as an adaptive disease caused by the lack of ability to deal with new computer technologies in a healthy way. Its conception and etymology can be traced to the 1980s with the publication of the book *Technostress: The Human Cost of the Computer Revolution* (Brod, 1984), which mainly pointed out the negative aspects of computer use. In a little over a decade, this understanding of technostress was nuanced by Weil and Rosen (1997, p. 5) to include “the negative impacts on attitudes, thoughts or behaviors, caused directly or indirectly through technology,” thus allowing a sufficiently broad definition of the phenomenon to incorporate successive technological advances.

In the Spanish sphere, Salanova (2003) considers technostress as:

A negative psychological state related to the use of ICT or a threat to its use in the future. This state is conditioned by the perception of a mismatch between demands and resources related to the use of ICTs leading to a high level of unpleasant psychophysiological activation and the development of negative attitudes towards ICT (p. 231).

From this definition, various theoretical models that try to specifically explain the emergence of stress related to the use of technology within organizations have been proposed, which are either based on general classical theories of stress or specifically arise to explain this phenomenon.

A transactional model of stress and coping defines psychological stress as “a particular relationship between the person and the environment that is considered as an imposition or an overcoming of their resources and endangers their wellbeing” (Lazarus, 1966; Lazarus and Folkman, 1984). Thus, technostress occurs when the competency requirements of information and communication technology (ICT) exceed the level of real user competence within an organization, or when technological demands exceed the resources or capacity to face them (Tarafdar et al., 2010, 2015; Ayyagari et al., 2011; Hung et al., 2011; Fuglseth and Sørebø, 2014; Yin et al., 2014; Galluch et al., 2015; Srivastava et al., 2015; Fischer and Riedl, 2017).

According to the job demands-resources model (Demerouti et al., 2001), each work environment has its own characteristics that can be divided into two general categories (i.e., work demands and work resources). Moreover, this model states that the health and wellbeing of employees are the result of a balance between positive (i.e., resources) and negative (i.e., demands) work characteristics. Applying this to the phenomenon at hand (Salanova et al., 2007; López-Araujo and Osca, 2008; Wang et al., 2020), the supporters of this theory explain that technostress results from high demands (i.e., techno demands) and lack of technological resources (i.e., techno resources) at work.

In addition, the person-environment misfit theory (P-E fit theory; Harrison, 1978; Edwards, 1996; Edwards et al., 1998) assumes that there is an equilibrium between people and their environment; when this relationship is out of balance, tension is generated (Ayyagari et al., 2011). Stress is caused neither by the person nor the environment, but appears when there is no adjustment between the two (e.g., between the needs of the person and the resources of the environment, or between the aptitudes and abilities of the person and the demands of the environment). Thus, technostress is conceptualized as a misfit between a person and the environment. It is not only limited by technology itself but also by the organization that has established the requirements for its use, and the members of the organization that on multiple occasions have an influence on the individual’s use of technology (Avanzi et al., 2018).

Most measurement instruments have focused on the subjective experience of technostress, dimensioning it in five different factors known as techno-invasion, techno-insecurity, techno-complexity, techno-uncertainty, and techno-overload (Tarafdar et al., 2007; Ragu-Nathan et al., 2008; Wang et al., 2008; Salanova et al., 2013; Chen, 2015; Alam, 2016; Chen and Muthitacharoen, 2016; Krishnan, 2017; Chandra et al., 2019). In this way, the objective perspective of the person-environment interaction, or the elements described in the previous theories, are being ignored. Thus, technostress is conceptualized as an imbalance between the technological skills of the subjects and the technological demands of the institution in which they work.

Despite the breadth of studies that focus on the negative consequences of technostress (Tarafdar et al., 2015; Hsiao, 2017) and its influence on work performance (Jung et al., 2012; Jena, 2015), practically all of these studies focus on administration and industry (Ragu-Nathan et al., 2008; Fuglseth and Sørebø, 2014; Marchiori et al., 2019). In general, these works ignored the rapid technological advances that have been incorporated in the field of education, and university education in particular, and has allowed today the use of new technologies for teaching online.

In the university context, Wang and Li (2019) have been the only authors that have corroborated the P-E fit theory as an explanatory model of technostress in a sample of university professors from five Chinese universities. This validated a measurement instrument specifically adapted for higher education and showed how dimensions of the aforementioned theory can affect the work performance of teachers.

Based on previous studies, the objective of this research is twofold:

1. To determine whether the theoretical perspective of the P-E fit theory explains the existence of technostress among Spanish university students.
2. To adapt the teacher technostress questionnaire by Wang and Li (2019) for Western university students and corroborate its psychometric properties.

## Materials and Methods

### Procedure and Participants

The adaptation of the Wang and Li (2019) technostress questionnaire for Spanish students began with a translation of the elements that make up the original scale into Spanish by specialized translators from the Official School of Languages at the University of A Coruña. After, the items were adapted for a university students population. In this process, references to teaching were replaced with academic tasks or studies (e.g., study and school work), while references to work centers were replaced with “university”; thus, avoiding references to paid work and replacing it with academic tasks carried out by students. To check its concordance with the original scale, the new scale was subjected to a reverse translation process (as per Barbero et al., 2008), to check the concordance of the new scale with the original.

After the first Spanish version of the instrument was obtained, the following procedure was followed:

First, specialists in research methodology and instrument construction analyzed the scale to check the extent of the effect that the changes might have had on the internal structure of the scale.

Second, the scale was administered to a group of 10 students who, with their answers and annotations, allowed us to see the extent of the scale’s appropriateness in language and representation.

This process of expert validation with the target group allowed the researchers to adjust any confusing terms. The Spanish version of the 22 items that make up the scale (Table A1) was then obtained and digitized using the online platform Microsoft Forms. Subsequently, the form was sent en masse by email to participating universities through their respective distribution lists. The 5 Likert-type response options of the original scale had been preserved in the new scale (1- strongly disagree; 2- disagree; 3- neither disagree nor agree; 4- agree; and 5- strongly agree).

The instructions sent via email described the research objectives, identified the authors, and assured the anonymity of answers provided. No personal data that would allow identification of the students were collected, thus complying with the requirements on the regulation of personal data of the ethics committees of the universities involved as well as the recommendations of the Declaration of Helsinki (2016/679), as approved by the European Parliament of the European Union.

Students were asked for their consent to participate in the study and were informed that their information was to be used solely and exclusively for the study. To accomplish this, a mandatory question was introduced prior to viewing the questionnaire that, if not answered in the affirmative, prevented the completion of the questionnaire.

Data collection began in mid-April (specifically on the 17th), coinciding with the month of confinement of the population in Spain due to the state of alarm decreed by the Government in response to the COVID-19 pandemic, and ended a month later (May 16), when the first deconfinement measures occurred.

During the period of data collection, all official in-person training activities in Spanish educational institutions (i.e., nursery schools, primary, secondary, high school, vocational training centers, and university education) were suspended and had to be carried out remotely or online.

The sample comprised 1,744 students (46.4% men and 53.3% women) who were studying at public (84.5%) and private (15.5%) universities in Spain. Their average age was 24.91 years, without significant differences according to gender. A total of 64.7% of the participants studied at a university center with an exclusive face-to-face teaching modality, while 35.3% of the students studied in a university with an online or blended teaching modality.

The average age of university students who carried out their studies online was significantly higher (28.71 years) than students who did so through the blended teaching modality (23.09 years); there was a statistically significant difference (*t* = 13.496, *p* < 0.01). With regards to their studies, 25.9% of the participants were in the first year of undergraduate studies, 22.7% in the second, 21.2% in the third, 19.9% in the fourth, and 2% in the fifth. Only 6.4% were studying a master’s degree, and 3% were studying for a doctorate. Also, 48.72% of the students chose a degree in the field of social and legal sciences, 25.26% in science, 16.58% in health sciences, and 6.81% in arts and humanities.

### Measures

To estimate the phenomenon of technostress among university students, we used the technostress questionnaire (Wang and Li, 2019) that was based on a multidimensional person-environment model. In our proposed instrument, technostress is conceptualized as the result of maladjustment in three main areas of people’s interaction with the environment in which they work: from person to organization (person-organization misfit; P-O), person to technology (person-technology misfit; P-T), and people to each other (person-person technology; P-P). The maladjustment of P-O and P-T was also conceptualized from two paths: on one hand, the lack of abilities of the subjects, and on the other, a lack of supplies to adapt to changes.

These dimensions were analyzed with university teachers. It is understood that these teachers are essential to the case at hand (university students), so their participation was considered, conceptualizing it as follows:

P-O encompasses both the maladjustment of the abilities of the subjects in relation to the new demands of their condition as students (abilities-demands misfit, A-D) as well as the lack of support or resources on the part of the institution in the face of the new needs of the students (needs-supplies misfit, N-S).

P-T assumes that the technological skills of students will quickly become obsolete due to the constant evolution in technological and information systems, forcing them to work faster and with greater technological demands (A-D). Likewise, inappropriate use of technology may originate from the use of technological tools that are inadequate for the task, or from a lack of customization of available tools (N-S).

P-P is conceptualized as the lack of support on the part of other students when carrying out academic tasks, which can increase the feeling of uselessness of new technologies and increase technostress.

### Statistical Analyses

An exploratory factorial analysis (EFA) following the Kaiser principle was carried out using principal components and varimax rotation. The Bartlett sphericity test statistic allowed us to estimate the adequacy of the factorial solution.

Once the main factors were identified through the EFA, a confirmatory factorial analysis (CFA) was carried out to determine the goodness of fit of the factorial structure. The estimation method used was unweighted least squares (ULS). To evaluate the adjustment value of the model, the following indices were used: goodness of fit index (GFI), adjusted goodness of fit index (AGFI), root mean square residual index (RMR), normed fit index (NFI), and relative fit index (RFI). ULS is used for variables that do not follow a normal distribution and is especially recommended when the variables are of the ordinal type (Morata-Ramírez et al., 2015). In accordance with authors such as Kline (2016), values show a good model fit if RMR ≤ 0.06 and GFI, AGFI, NFI, and RFI > 0.90.

To ascertain the psychometric properties of the questionnaire, a reliability analysis was performed by calculating the Cronbach’s alpha and Omega reliability coefficients. All analyses were performed using the IBM SPSS statistical software (version 25), AMOS extension for SPSS, and the free-access computer program “R” version 3.1.2 (R Development Core Team, 2007).

## Results

### Exploratory Factor Analysis

The EFA, using the varimax method and based on an eigenvalue greater than 1, showed very good statistical results (KMO = 0.950, *df* = 231, *p* < 0.001), with an explained variance percentage of 60%; the three factors clearly differentiated between the proposed general theoretical areas (see Table 1).

**TABLE 1:** Exploratory factor analysis based on an eigenvalue greater than 1.

	1	2	3
ADO1	0.777		
ADO2	0.738		
ADO3	0.736		
ADO4	0.707		
ADO5	0.710		
NSO1		0.736	
NSO2		0.756	
NSO3		0.772	
NSO4		0.713	
ADT1	0.637		
ADT2	0.590		
ADT3	0.769		
ADT4	0.677		
NST1		0.651	
NST2		0.649	
NST3		0.619	
NST4		0.665	
NST5		0.672	
PPF1			0.782
PPF2			0.722
PPF3			0.660
PPF4			0.516
% of variance	28.94	18.27	13.25

In this way, a first factor was observed, which was called the A-D factor. This includes items that indicate an imbalance between the skills of the subjects and the demands of the situation in which they carry out their work. This imbalance can come mainly from two sources: the demands that come from the organization (i.e., the university) and demands of the use of technology (i.e., those that come from the computer programs used).

The second factor grouped all the needs that users have and the needs that have not been covered, thus generating a feeling of stress. These grouped items indicate that this imbalance can come from the institution itself (i.e., the university), as well as from student needs and proper technological resources.

Finally, the third factor referred to as the human factor, grouped the behaviors and responses of students regarding the use of technology within the university.

In the factorial model obtained, two items with moderate factor weights (NST1 and NST2) were observed in a different factor than what would be expected, depending on the theoretical model proposed.

If the factorial solution is forced into five factors, following the model proposed by Wang and Li (2019), the good statistical results of the three-factor solution are maintained (KMO = 0.950; *df* = 231; *p* < 0.001), with a slight increase in the explained variance that stood at 68%, but observing the same saturation problems in items NST1 and NST2.

The identification in both analyses of these two items with moderate weights, in a factor different from what might be expected based on the proposed theoretical model, advised their elimination; a decision that was supported by theoretical reasons, in addition to the statistical criteria. The wording of these two items broke the general response dynamic: in general, the respondents were asked to position themselves (e.g., “my university does not provide me,” “my university does not train me,” etc.), but for these two items, they were asked opinions about the role played by the ICT in their university (e.g., “ICT in my university is not effective” and “ICT in my university is not relevant”).

After these items were eliminated, another EFA was carried out, obtaining a factorial solution of five factors, with an explained variance percentage of 70% (KMO = 0.947, *df* = 190, *p* < 0.001; see Table 2).

**TABLE 2:** Exploratory factor analysis (5-factor solution).

	1	2	3	4	5
ADO1	0.747				
ADO2	0.707				
ADO3	0.793				
ADO4	0.759				
ADO5	0.707				
NSO1		0.802			
NSO2		0.804			
NSO3		0.818			
NSO4		0.643			
ADT1			0.508		
ADT2			0.568		
ADT3			0.743		
ADT4			0.593		
NST3				0.819	
NST4				0.734	
NST5				0.749	
PPF1					0.895
PPF2					0.838
PPF3					0.773
PPF4					0.628
% of variance	24.87	16.01	13.19	10.95	5.65
% of accumulated variance	24.87	40.87	54.06	65.01	70.66

### Confirmatory Factor Analysis

Following the EFA, the two factor models resulting from the elimination of the items NST1 and NST2 were tested using confirmatory factor analysis.

The results obtained from the confirmatory factor analysis with the three-factor solution showed an unacceptable RMR index based on the proposed standards (RMR = 0.07), which meant that the theoretical model obtained had to be discarded.

The factorial solution of five factors was the solution that obtained the best statistical results based on the established requirements (RMR ≤ 0.06 and GFI, AGFI, NFI, and RFI > 0.90), with a goodness of fit that showed the robustness of the model (see Table 3).

**TABLE 3:** Results of the confirmatory factor analysis.

	Three-factor solution	Five-factor solution
RMR	0.080	0.054
GFI	0.980	0.995
AGFI	0.980	0.994
NFI	0.980	0.994
RFI	0.978	0.993
χ^2^	3654.825	1282.370

**FIGURE 1:**
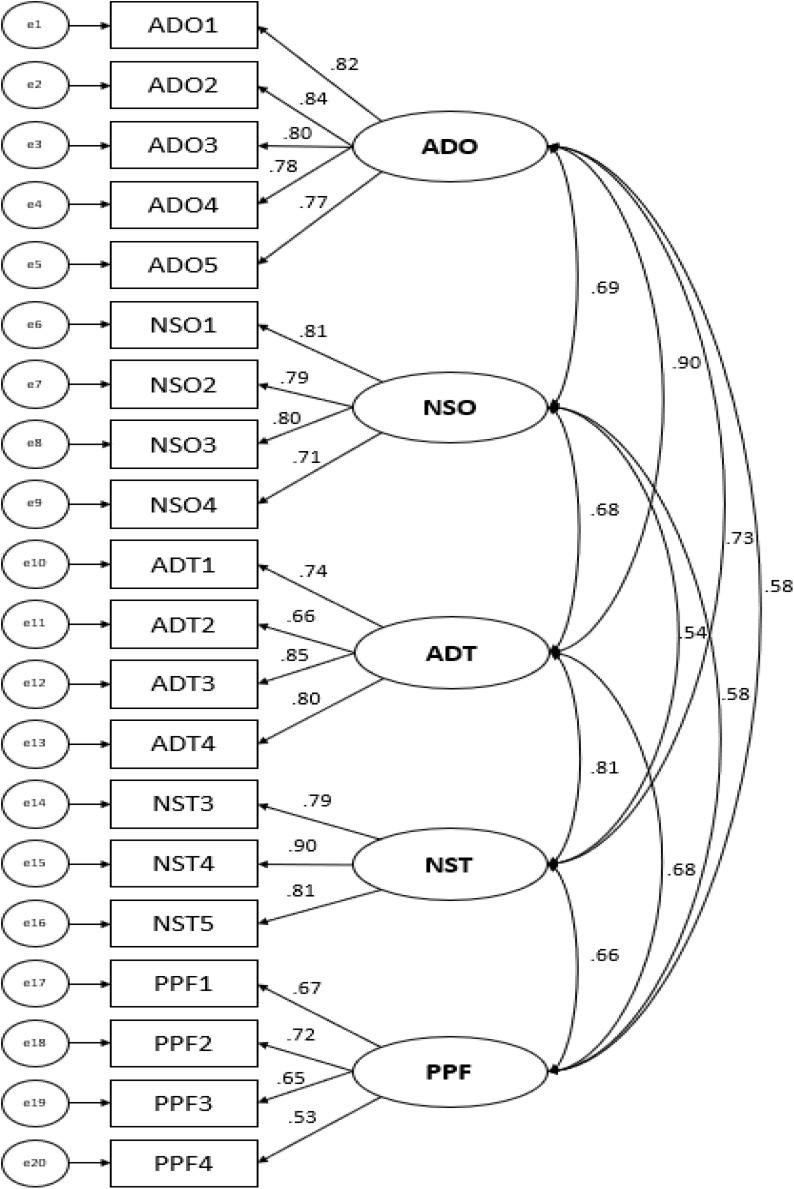
Confirmatory factor analysis.

As shown in Figure 1, the correlations ranged from 0.90 (organizational and technological abilities-demands) to 0.54 (organizational and technological needs-supplies). The factor weights of the items that made up each of the factors were high (in most cases exceeding 0.70), except when considering the interpersonal interaction factor (person-person), where the factor weight of the last item did not reach the aforementioned cut-off point (see Figure 1).

To corroborate the obtained results, a cross-validation was carried out from a random segmentation of the sample according to gender. A subsample of women was used to verify the results obtained from the EFA, while a subsample of men replicated the theoretical model proposed for the confirmatory factor analysis. The results of the EFA with the subsample of women replicated the factorial structure formed by the five factors obtained in the entire sample, which explained 70.74% of the variance obtained for women (KMO = 0.945, *df* = 190, *p* < 0.001). While that carried out with the subsample of men, identified a root mean square residual index that was considered acceptable (RMR = 0.06), with good results in the rest of the goodness-of-fit indices (GFI = 0.995; AGFI = 0.993; NFI = 0.993; RFI = 0.992).

To calculate the unidimensionality of the instrument, the analyses were carried out from the matrix of polychoric correlations, obtaining some results practically identical to those obtained in the confirmatory factor analysis. The correlations ranged from 0.896 (organizational and technological abilities-demands) to 0.536 (organizational and technological needs-supplies), with intermediate scores in the rest of the factors analyzed (ADO-NSO = 0.692; NSO-ADT = 0.682; ADT-NST = 0.810; NST-PPF = 0.657; ADO-NST = 0.733; ADO-PPF = 0.580; NSO-PPF = 0.581; ADT-PPF = 0.676).

### Reliability Analyses

The reliability analysis for the entire scale showed a Cronbach’s α of 0.942, which was considered excellent (Hinton et al., 2004), and this meant all items contributed significantly to the overall result.

Meanwhile, the reliability analysis for the final proposed reduced twenty-item scale showed a Cronbach’s α of 0.939. Considering the type of teaching modality (face-to-face or online), it was observed that the reliability results improved in the sample of university students who carried out their studies online (α = 0.943) compared with students who did so face-to-face (α = 0.935).

Since the scale items were ordinal, and to obtain a more accurate measurement of the reliability of the instrument, the Omega reliability coefficient was calculated, which confirmed the good results obtained (Ω = 0.96), and far exceeded the acceptable values established by other authors (Campo-Arias and Oviedo, 2008).

The reliability analysis for the factors that made up the reduced scale also showed good reliability, with high values in all the constructs that needed to be measured (see Table 4).

**TABLE 4:** Reliability analysis of the factors and psychometric properties of the technostress questionnaire.

	α	*M-i*	*SD-i*	*r^*c*^_*i*_-t*	α-*i*
**ADO**	0.901				
ADO1		11.74	19.497	0.784	0.872
ADO2		11.83	19.591	0.793	0.871
ADO3		12.11	19.501	0.764	0.877
ADO4		12.28	20.202	0.720	0.886
ADO5		11.40	19.476	0.710	0.889
**NSO**	0.859				
NSO1		9.03	10.421	0.747	0.802
NSO2		8.80	10.546	0.737	0.806
NSO3		8.82	10.396	0.752	0.800
NSO4		9.05	11.309	0.586	0.868
**ADT**	0.847				
ADT1		8.27	11.937	0.610	0.838
ADT2		9.10	12.157	0.641	0.823
ADT3		8.83	10.908	0.778	0.764
ADT4		8.96	11.444	0.713	0.793
**NST**	0.872				
NST3		5.08	5.485	0.766	0.810
NST4		4.97	5.460	0.780	0.797
NST5		5.13	5.666	0.720	0.852
**PPF**	0.736				
PPF1		7.78	8.050	0.613	0.630
PPF2		7.42	7.844	0.587	0.641
PPF3		7.58	7.927	0.539	0.669
PPF4		6.95	8.800	0.388	0.756

*Item factor-correlation (r^c^_i_-t), Cronbach’s alpha if item deleted (α-i), mean if item deleted (M-i), standard deviation if item deleted (SD-i).*

## Discussion

An adaptation of the Wang and Li (2019) questionnaire was carried out through its translation into Spanish and the adjustment of the items to the new study population, obtaining a twenty-item scale to measure technostress in Spanish university students.

The results obtained in the validation process showed excellent psychometric results and a factor structure that indicated that technostress can be conceptualized within the theory of person-environment interaction, as a product of the imbalance between demands (abilities) and resources (needs), in addition to being influenced by the behavior of other students.

The conceptualization of technostress observed by Wang and Li (2019) for Chinese university teachers was maintained in a population as different as the Spanish university students, in that it can be seen that technostress is a multidimensional process where it is observed as well as, for the sample of teachers, the interaction mismatches between a person’s abilities and the organization’s demands in relation to the use of technology (abilities-demands organization, ADO), person’s abilities and the demands of the technology itself (abilities-demands technology, ADT), needs of the person and the resources that the organization makes available to them to carry out their tasks (needs-supplies organization, NSO), needs of the person and their own available technological resources (needs-supplies technology, NST), and influence of the interpersonal factor or relations between fellow students regarding the use of technology in their role as students (person-people).

The reliability analysis obtained for the final proposed reduced scale of twenty items maintained the good statistical results of the original scale applied to university teachers and preserved the factor structure obtained in the same population. With the reduction of items that make up the needs-supplies misfit factor (in P-T misfit), the reliability results obtained for this factor increased in relation to the original validation of the instrument.

According to the estimates made by Wang and Li (2019), for the teaching version of the technostress scale, a Western adaptation of the technostress scale for university students would consist of twenty items (with a total possible scores ranging from 20 to 100), indicating that the higher the score, the higher the level of technostress. Specifically, a score of 20 would indicate the absence of technostress, a score of 21–60 a mild level of technostress, a score of 61–80 a moderate level of technostress, and a score > 81 a severe level of technostress.

Moreover, this study addressed the limitations of an initial adaptation and validation of this instrument among Chinese university students (Wang et al., 2020), where the authors pointed out the need for the scale to be validated from the Western cultural context, using a more balanced sample of men and women and a greater representative sample of different universities.

Overcoming the limitations described above, a validation of the technostress questionnaire was carried out for Western university students, starting from the version already validated among university teachers, which allowed for a more complete view compared with the process of validation of the technostress questionnaire among Chinese university students (Wang et al., 2020).

The results obtained in the present study offer a more complete view of the phenomenon of technostress among university students with a 20-item scale that differentiated skills and resources, both organizational (university) and technological, and also incorporated the interactions that can occur between students when using technology in the educational environment.

Undoubtedly, these data may represent more than one element of the feeling of technostress, which allows for a more complete view than that obtained by the authors (Wang et al., 2020) in the process of developing and validating their technostress scale among university students, which only considered the technological dimension of these factors (ADT, NST), excluding the organizational and interpersonal interaction aspects that can influence the technological stress process.

Despite the results obtained, this study has some limitations that must be considered when assessing the representativeness of the results.

First, a cross-sectional design was adopted to obtain the data, which does not allow for the establishment of causal relationships; thus, it is necessary to carry out longitudinal studies to assess such relationships.

Second, the data were obtained by means of a self-evaluation questionnaire. Hence, it may be interesting to use objective data to contrast the information provided by the subjects themselves.

Third, different variables such as the type of university (public-private) or geographical location could improve the representativeness of the study and the validity of the questionnaire.

Finally, the data collection was carried out during a period of special stress among the participants, such as the compulsory use of new technologies for the continuation of their studies during the period of confinement necessitated by the COVID-19 crisis, the instrument should be tested once this situation has passed to verify the stability of the results.

## Data Availability Statement

The raw data supporting the conclusions of this article will be made available by the authors, without undue reservation, to any qualified researcher.

## Ethics Statement

Ethical review and approval was not required for the study on human participants in accordance with the local legislation and institutional requirements. The patients/participants provided their written informed consent to participate in this study.

## Author Contributions

All authors listed have made a substantial, direct and intellectual contribution to the work, and approved it for publication.

## Conflict of Interest

The authors declare that the research was conducted in the absence of any commercial or financial relationships that could be construed as a potential conflict of interest.

## Appendix

**TABLE A1:** Original and adapted scale used in the adaptation of the instrument to Spanish students.

Constructs	Items of the original version of the instrument (Wang and Li, 2019)	Items used in the Spanish version
ADO	I find it difficult to meet the high demands of school policies regarding the use of ICTs at work.	1. Me resulta difícil satisfacer las altas demandas de mi universidad, con respecto al uso de las TIC
	I find it difficult to effectively implement school policies regarding the use of ICTs at work.	2. Me resulta difícil implementar con eficacia las indicaciones de mi universidad, sobre el uso de las TIC
	My current capability is insufficient to implementing school policies regarding the use of ICTs at work.	3. Mi capacidad actual es insuficiente para implementar las indicaciones de universidad, sobre el uso de las TIC.
	My current skillset is insufficient for the successful implementation of school policies regarding the use of ICTs at work.	4. Mis habilidades actuales son insuficientes para implementar las indicaciones de mi universidad, sobre el uso de las TIC.
	I find it hard to adjust my current work pattern so as to comply with school policies regarding the use of ICTs at work.	5. Me resulta difícil ajustar mi patrón de estudio actual para cumplir con las indicaciones de mi universidad, sobre el uso de las TIC.
NSO	My school does not provide me with sufficient professional training to effectively use ICTs at work.	6. Mi universidad no me brinda suficiente información para usar las TIC de manera efectiva en mi trabajo como estudiante.
	My school does not provide me with sufficient incentives to effectively use ICTs at work.	7. Mi universidad no me brinda incentivos suficientes para utilizar las TIC de manera efectiva en mis actividades como estudiante.
	The professional training provided by my school is not very relevant for the effective use of ICTs at work.	8. La información facilitada por mi universidad no es muy útil para el uso efectivo de las TIC.
	I do not have a culture in my school that encourages the use of innovative tools such as ICTs at work.	9. No tengo una cultura en mi universidad que fomente el uso de herramientas innovadoras como las TIC.
ADT	I feel pressured to effectively use ICTs at work.	10. Me siento presionado para usar las TIC de manera efectiva en mis trabajos universitarios.
	I find it difficult to effectively use ICTs due to my limited investment of time and effort.	11. Me resulta difícil utilizar las TIC de manera efectiva debido al poco tiempo y esfuerzo que le dedico
	I find it difficult to cope with the high demands of ICTs with my current capability.	12. Me resulta difícil hacer frente a las altas demandas de las TIC con mi capacidad actual.
	I find it difficult to catch up with the rapid changes of ICTs with my current skillset.	13. Me resulta difícil ponerme al día con los rápidos cambios de las TIC.
NST	The ICTs in my school are not effective in helping me increase my productivity at work.	14. Las TIC en mi centro educativo no son efectivas para ayudarme a aumentar mi productividad como estudiante.
	The ICTs in my school are not very relevant for the improvement of my work.	15. Las TIC en mi universidad no son muy importantes
	I am irritated by the vast variety of ICTs that are utilized in my school.	16. Estoy irritado por la gran variedad de TIC que se utilizan en mi universidad
	The various ICTs complicate my decision-making process at work.	17. Las diversas TIC complican mi proceso de toma de decisiones
	I am annoyed by the excessive use of ICTs in my school.	18. Me molesta el uso excesivo de las TIC en mi universidad
PPF	I do not have sufficient support from my colleagues for the use of ICTs at work.	19. No tengo el apoyo suficiente de mis compañeros para el uso de las TIC.
	My colleagues are not encouraging with regard to the innovative use of ICTs at work.	20. Mis compañeros no son positivos con respecto al uso innovador de las TIC en mi universidad
	I do not have a team to collaborate with so as to figure out an effective way to use ICTs at work.	21. No tengo un equipo con el que colaborar para encontrar una forma eficaz de usar las TIC en en mi trabajo como estudiante universitario.
	I often feel that I am alone in exploring the innovative use of ICTs at work.	22. A menudo siento que estoy solo explorando el uso innovador de las TIC.
